# MINFLUX: μs and nm precision 3D tracking of dynamic lipid mobility on nanoparticles†

**DOI:** 10.1039/d5nr00948k

**Published:** 2025-07-02

**Authors:** Laurence W. Fitzpatrick, Laura Woythe, Ziqiang Huang, Sebastian Schnorrenberg, Timo Zimmermann, Lorenzo Albertazzi

**Affiliations:** a Department of Biomedical Engineering, Institute of Complex Molecular Systems, Eindhoven University of Technology Eindhoven The Netherlands L.albertazzi@tue.nl; b EMBL Imaging Centre, EMBL Heidelberg Germany

## Abstract

MINFLUX microscopy opened unique opportunities to investigate complex molecular systems at a single molecule level with μs temporal and nm spatial resolutions in 3D. Here, we demonstrate its use for the characterization of nanomaterials, providing a framework for MINFLUX imaging and data analysis tailored to understanding the complex dynamics of nanosized Silica-Supported Lipid Bilayers (SSLBs).

## Abstract 2

MINFLUX microscopy opened unique opportunities to investigate complex molecular systems at a single molecule level with μs temporal and nm spatial resolutions in 3D.

## Graphical abstract

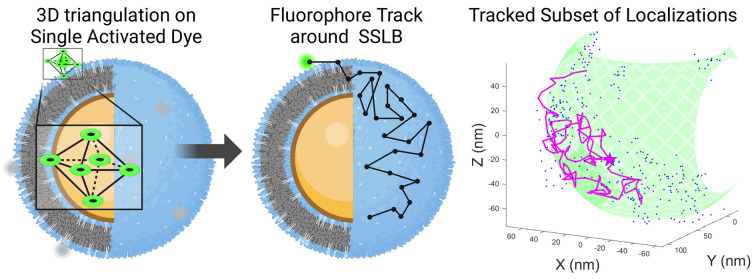

MINFLUX^1^ (minimal fluorescence photon fluxes microscopy) is currently the optical imaging method with the highest temporal and spatial resolutions (<5 nm & 10's μs). In MINFLUX a donut-shaped excitation beam is scanned around a molecule of interest to calculate the position of the molecule in 3D based on the fluorescent signal that was emitted for the individual scanned positions. This “triangulation” procedure allows MINFLUX to locate the molecules positions resulting in two key advantages: (i) the molecule of interest can be localized and tracked with extremely high resolution and (ii) it minimizes bleaching as the molecule is close to the center of the donut shaped beam which features zero-intensity allowing it to be tracked for very long times. This represents a paradigm change in fluorescence imaging: the position of a molecule is precisely determined not by localizing it but rather detecting its influence in the surrounding volume, minimizing the photons needed for localization. Introduced in 2017, it has been applied to biological systems,^2,3^ specifically to image and track intracellular locations and processes,^2–8^ such as nuclear pores, lipid diffusion, clathrin, and even individual proteins, such as kinesin-1 motion.

Yet MINFLUX holds vast potential across a range of applications beyond biology, of which nanomaterials are currently unexplored. Here we report the application of MINFLUX for the first time to study nanomaterials, explicitly the inherent dynamic lipid mobility within Silica-Supported Lipid Bilayers (SSLB). SSLBs have been applied to a range of applications, such as drug delivery systems, due to their inherent bio-compatibility and the tunability of the lipid bilayer over the solid silica core in terms of fluidity, dynamics, and temperature-responsivness.^9–14^ Lipid mobility is of great interest for several biomedical applications as it allows surface ligands (*e.g.*, targeting moieties) to be dynamic on the particle surface and adapt or reconfigure themselves for target binding. In this context it is crucial to assess the inherent lipid mobility and provide a quantitative estimation of their motion.

Previous Fluorescent Response After Photobleaching (FRAP)^11,15^ and molecular dynamic simulations^16^ looked at lipid diffusion on flat surfaces or around microparticles, placing either the bulk diffusion, or an expected value of single lipid mobility within such layers around 1–10 μm^2^ s^−1^. These yield valuable information, however, FRAP cannot be applied to nanoparticles due to its lack of resolution (both spatially and temporally), whilst diffusion properties of lipids on nanoscale substrates are still unknown. Moreover, FRAP provides only bulk data missing the heterogeneity within and between lipids and other rare events. To this end, this is the first attempt to image and track individual molecules within such a nano-system bridging between bulk FRAP investigations and the more detailed simulations. The acquired results on two different Silica (Si) SSLB systems, namely one coated with Dioleoyl phosphatidylcholine (DOPC), and another with Dipalmitoyl phosphatidylcholine (DPPC), indicate that MINFLUX is ideal for studying these systems, giving new information at a single molecule level across a number of particles, in line with previous studies.^11,15–21^

A schematic representation of the MINFLUX experimental system is presented in Fig. 1, with a sample result from a Si-DOPC SSLB also shown. We previously stated that these systems are interesting for drug delivery as the mobility of the lipids may allow for the adaption of the ligand distribution to the target cell, but it was previously impossible to measure such mobility before due to the lack of suitable techniques. Particles were physisorbed on a glass slide and a sub-set of the lipids within the SSLB was labelled with a photoactivatable fluorophore, CAGE 635 (Abberior). This fluorophore is a rhodamine caged with a diazo-indanone group that is cleaved with UV irradiation, releasing nitrogen and restoring its fluorescence in an irreversible fashion.^22^ A low UV irradiation power was used in order to be sure to activate only one molecule per particle at the time (Fig. 1B). Once uncaged the Targeted Coordinate Pattern (TCP) begins to focus in on it (Fig. 1C). The microscope triangulates the donut-shaped beam around the molecule and if the collective response over the dwell time is above a fixed photon threshold, the position of the molecule is calculated with a certain precision. This process is re-iterated, tracking the fluorophore in 3D, stopping only once the emission from the fluorophore is no longer detectable (Fig. 1D). Within the samples of interest, this results in a 3D point cloud of recorded averaged localizations that relate to the movement of the labelled lipid. An example of such a point cloud (Fig. 1E) shows that the localizations allow the reconstruction of the underlying particles size and shape with nanometric precision. Furthermore, the point-to-point tracking (Fig. 1F) yields diffusion coefficient, speed, and confinement quantifications.

**Fig. 1:**
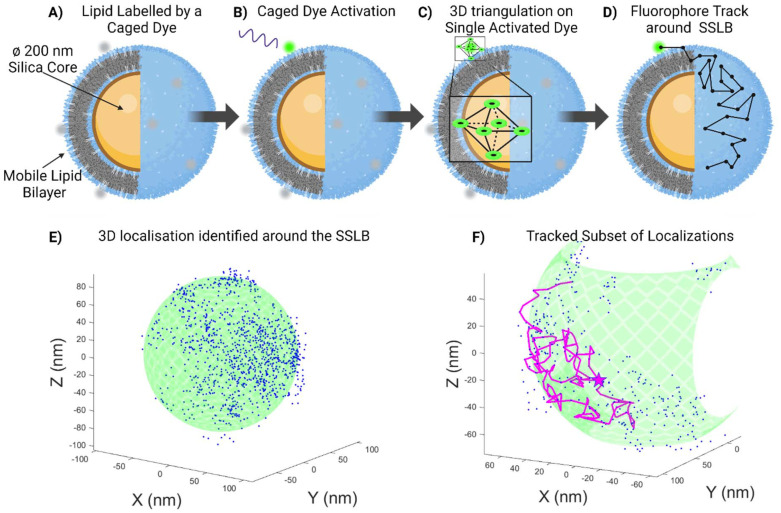
Not to scale schematic representations of (A) the mobile silica-supported lipid bilayer (SSLB), with a subset of the lipid bilayer labelled with a caged dye, which is then activated by UV radiation (B). 3D MINFLUX utilizes a triangulation called a Targeted Coordinate Pattern (TCP) *ca.* 70–100 nm in size (C) to locate the single fluorophore, which enables single molecule tracking and the path around the SSLB shows the freedom of movement of the lipid around the SSLB (D). Experimentally acquired data (E), showing the acquired localization around a single SSLB with the expected Silica Core location (green) displayed for illustrative purposes. A subset of the tracking is shown colored in magenta (F) to show the freedom of movement of this particular lipid within this particular domain.

An extensive custom analysis pipeline^23^ including a GUI was developed with the purpose to assess multiple aspects of the acquired MINFLUX localizations (Fig. 2), which aims to visualize the raw data together with the results of the post-acquisition data assessment employed. We believe that this may simplify the data analysis for non-experts and make MINFLUX imaging more accessible to the nanotechnology community. Therefore to facilitate the access of MINFLUX to all the community we also made this script freely available.^23^ The GUI automatically plots raw data (localization map, particle size, velocity, and time intervals), the filtered (>100 photon) and the processed data, to display parameters like MSD and diffusion coefficients.

**Fig. 2:**
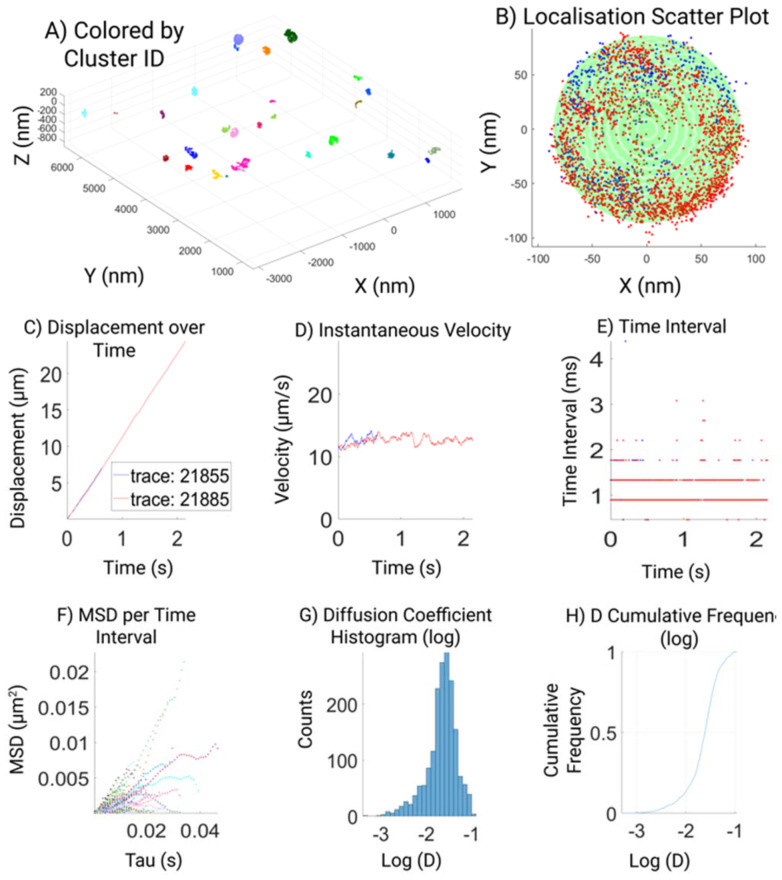
Data visualizations with the custom-made GUI tool, showing representative Si-DOPC data over, the total area of acquisition (A), and the localization around a single identified SSLB with two tracks around the core (B). The displacement over time (C), the instantaneous velocity (D) and time intervals (E) of the raw data tracs shown in (B). Finally, the MSD per time interval (F), the histogram of diffusion coefficients (G) and the cumulated frequency of acquire diffusion coefficients (H) of the same data post filtering.

This custom data processing and analysis workflow is explained here and facilitates the analysis of the lipid mobility on silicon nanoparticles. Trajectories from fluorescent molecules are pre-processed and reconstructed from MINFLUX data, to quantify the underline diffusion properties, as a readout of the lipid mobility. The pre-processing, reconstruction, and visualization of the MINFLUX tracking data are all done through custom written MATLAB scripts and GUI tools.

In the first pre-process step, we performed a set of filtering to the MINFLUX raw data to remove noise and data that was not suitable for processing. We start with the raw data that was exported to MATLAB data (.mat) format. MINFLUX gathered tracking yields groups with an assigned unique ID to each track, denoted as ‘tid’. The ‘tid’ attribute is assigned to every localization as part of the raw data. Therefore, we first extracted 3D coordinates, the associated time stamp, and associated ‘tid’ of all valid localizations from the raw data for pre-processing.

The pre-processing on the raw data consists of filtering and then a clustering step.

Spatial filtering was done on the localization data, by removing data located near the border (within 1% of the border) of the XY field of view. To ensure the completeness of tracking data at this stage, the filtering is at the track level. It means if any data point falls into the 1% marginal region, an entire track containing that data point is discarded.

At this stage, a refractive index mismatch^24,25^ (RIM) correction is also performed, to compensate for the axial aberration. To do this, we measured and equalized the spatial spread in each axis for every track. For a given trace, we compute first the interquartile range (iqr) between 25% and 75% percentile of *X*, *Y*, and *Z* axis of the data. And then we calculate the ratio of *XY* geometrical mean over *Z* iqr value, as the RIM score for each track. Finally, we calculated a weighted mean of the RIM scores from all the tracks, based on the number of data points within each track, to generate the final RIM correction factor. The RIM correction factor is measured on a daily basis and applied to all tracking experiments in the same day. For the 3 consecutive days of imaging, we measured the RIM correction factors as 0.6232, 0.6388, and 0.6237 (Fig. 3), indicating rather low variation of the axial aberration across days of imaging.

**Fig. 3:**
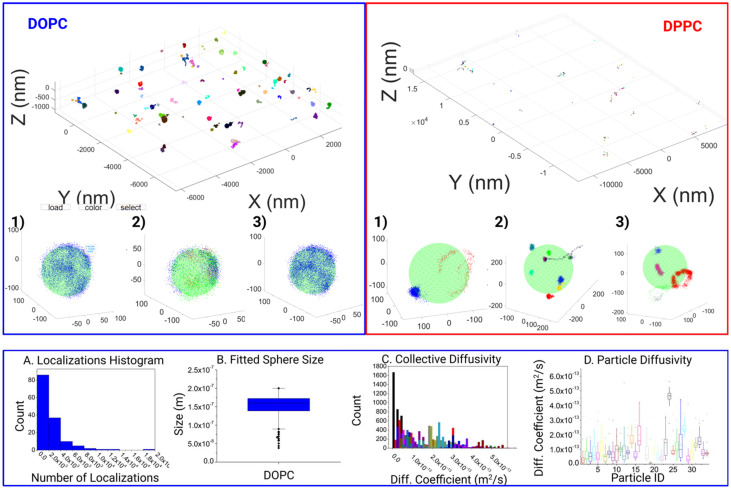
Full experimental field of view displaying final filtered and clustered data. Displaying a DOPC (left), and a DPPC (right) field. The stark difference in the distribution of responses is apparent and attributed to the increased confinement within the DPPC structure. Furthermore, variations within the responses can clearly be seen from 3 examples taken within the respective fields of view (1–3). Within the DOPC system, localizations per track can run into the thousands (A), the dataset is capable of yielding the fitted inner particle size of *ca.* 150 nm (B), with thousands of corresponding datapoints for the various characteristics such as diffusion coefficient (C) showing a wide variance even between particles (D).

Each MINFLUX tracking process takes roughly tens of data point to stabilize. As a result, tracks can exhibit a short tail-like portion in the beginning which reflects this initial targeting process. A given track ends normally when the tracking process slowly loses the currently tracked molecule, which can result in relatively low-quality data at the end of tracks. Therefore, for each track we discarded 100 data points both from the beginning and at the end of the track. By visual inspection, this filtering procedure effectively removes the ‘tail-like’ portion of each track. Shorter tracks that contain no more than 200 data points are discarded at this step.

The time stamps for each localization were exported with effective precision in the range of tens of microsecond. We extracted time intervals as the increment between time stamps of two adjacent localization events and rounded it to 0.1 millisecond (ms) precision for the MSD computation and diffusion analysis for this study.

After the filtering step, we then spatially clustered the processed data with a density-based scan (DBSCAN). We make use of the track identity of the data, to cluster on the track level, rather than individual localization data points. This way, the clustering process is sped up significantly, while the computation workload is greatly reduced. We first calculated the centroid coordinates of all tracks. Then all the centroid coordinates are clustered such that any pair of track centroids located within a radius of 200 nm to each other will be clustered together. A cluster ID is then assigned to each data point, similarly to the track ID. Naturally, data belonging to the same track also belongs to the same cluster.

We then fit a spherical shell to the clusters with the least square method. This fitting approach minimizes the residual sum of squared distance (error) of all points to the sphere surface. If the fitting error is too large, or the fitting result deviates enough from expected geometry (*e.g.*: points form a plane rather than a 3D spherical surface), the fitting is considered failed. Clusters with failed fitting are marked but kept for down-stream diffusion analysis. This is because when two or more silicon cores are close to each other, nanoparticles imaged on their surface sometimes cannot be distinguished by spatial clustering. Such cases would result in a cluster with failed sphere fitting but still contain meaningful data that can be visually categorized and further analyzed. Nevertheless, successful fitting results are also marked, and better fitting results with smaller fitting errors are sorted more to the front as good candidates for further inspection and analysis.

To analyze diffusion behavior, we compute mean squared displacement (MSD) and diffusion coefficient for each cluster. As mentioned previously we extracted time intervals (d*t*) between each adjacent localization and assessed this. It is obvious that the time intervals from MINFLUX tracking experiment are not always consistent, and instead roughly segregated into different levels (Fig. 2E). These levels are in fact correspond to the 1, 2, 3, or more rounds of MINFLUX beam pattern scanning on the tracked molecule. Since more rounds of scanning are made only when previous round(s) failed to locate the molecule, larger time interval is also normally associated with larger uncertainty in localization. To account for this, we break a filtered complete track into shorter track segments, around these large time intervals. We define a breaking criterion that only allows a maximum of 2 rounds of scanning within a given track segment. This is implemented by estimating the base level time interval minDt, which corresponds to only 1 round of scanning. We then set a threshold to the time intervals as 2.5 × minDt, to be the breaking criterion. This proven to be sufficient to effectively distinguish between 2 and 3 rounds scanning in the data. The track segments from all tracks belonging to a given cluster are stored and used in subsequent MSD computation.

To compute the MSD associated with each unique time interval, we used a modified version of msdanalyzer (https://tinevez.github.io/msdanalyzer), a designated MATLAB class to perform MSD computation and analysis. The main advantage of this package is it can deal with non-equidistant time intervals, which is better suited for this type of MINFLUX tracking data. We adopted a vectorized approach to save CPU time for the MSD computation in MATLAB, at the expense of RAM. With our code, the memory required to process a complete track segment consisting of ∼40 k data points would be roughly 128 Gb, as assessed on a Windows 10 machine with MATLAB 2023b. However, given the average time interval from MINFLUX tracking data is in the range of several hundreds of microseconds, we haven't encountered any complete track segments containing more than 10 k data points so far. In addition, for the objective of this study, we are mainly interested in the fast component of diffusion behavior and as a result we would not necessarily need the MSD computed from such long track segments. For each track segment extracted in the last step, all possible time intervals, d*t*, are calculated. Then MSD values corresponding to each unique d*t* values are computed. The MSD and d*t* pairs are computed and stored for each track segment. We also computed cluster-wise weighted average values, with the same procedure that is described in msdanalyzer. Shortly paraphrasing, the weights are taken to be the number of averaged delay (time interval), which favors short delays.

The diffusion coefficient can be calculated for each pair of MSD and d*t* as *D* = MSD/(2 × ndim × d*t*), with ndim being the dimensionality of the trajectory. Therefore, in the 3D tracking case, diffusion coefficient *D* is thus calculated as MSD/(6 × d*t*).

The analysis results from the above filtering, clustering, sphere fitting, and MSD analysis steps are all stored and exported to MATLAB data file format. The result file can be loaded into MATLAB for data visualization and further analysis, or to be loaded with the custom data visualization tool that is created together with the analysis scripts.

To facilitate data visualization and further analysis, a GUI tool which generates MATLAB figures is also created, as part of the analysis workflow (Fig. 2). The first overview figure displays coordinates of the processed data as a 3D scatter plot. It enables data load, coloration, and data selection through custom designed buttons (Fig. 2A). The ‘color’ toggles between 3 different color modes of the scatter plot: colored by track, colored by cluster, or colored by selected cluster, which is displayed in a second cluster view figure. The cluster view figure shows the selected cluster as a 3D scatter plot and uses different colors to differentiate different tracks belonging to the same cluster. If the sphere fitting was successful, a semi-transparent sphere shell is generated based on the fitting parameters and overlay with the scatter plot (Fig. 2B). We made 6 subplots to further display the properties of the tracking data and tracking result. The 6 subplots are gathered in 2 groups: the top 3 subplots show displacement, velocity, and time-intervals against time; the bottom 3 subplots show the MSD *vs.* time interval plot, histogram of the diffusion coefficient, and cumulative frequency of the diffusion coefficient. A time slider is also implemented to highlight tracking data and values (in the top 3 subplots) corresponding to the slider indicated time point.

Fig. 2 shows a typical output for a DOPC SSLB. Our analysis shows the overview of all the particles in the field of view that are clustered for further analysis (2A) and highlighting one selected particle and its tracks in different colors (2B). It is clear how single trajectories can feature hundreds of localizations due to minimal bleaching and can span the whole nanoparticle showing no confinement of DOPC as expected. With enough positions within the distribution of recorded localizations the size of the NP can be inferred by a sphere fitting (green shape). Then displacement (2C), velocity (2D), and time interval (2E), are extracted and plotted for quality readout. Finally, 2F–H shows the analysis of assessed diffusion coefficient (D), based on the highest quality filtered data.

For micro-scale or bulk lipids, the literature values for diffusion coefficient range from 1–10 μm^2^ s^−1^.^15,18,26,27^ Whilst, here in the example shown the Diffusion Coefficient for the choice of lipids is around 0.25 μm^2^ s^−1^. There are no comparable measurements on nanoscale-systems and theoretical prediction assumes the diffusion coefficient to decrease for smaller particles, which is in line with our observations. The difference can also be potentially due to the difference in temporal resolution between nanosecond simulations and sub millisecond experimental observations. Furthermore, we want to also highlight the limitations of MINFLUX as per the acquisition method and quality assessments, it is possible to lose tracks of fast molecular motion. Depending on the purpose, different quality thresholds can be applied reaching different trade-offs between data quality, precision, and speed.

With this tool, we moved on to analyze the differences in motion between DOPC and DPPC SSLB, two standard systems used in the literature. In all 144 individual clusters for DOPC SSLB's and 243 individual clusters for DPPC SSLB's were imaged and analyzed, and the results are shown in Fig. 3. A selection of both the Si-DOPC and Si-DPPC results from the GUI are given in the ESI (Fig. S1†), showing the heterogeneity of both systems. Naturally, DOPC should exist as a mobile phase, whilst DPPC is more gel like. As shown in Fig. 3, across the DOPC samples, localizations can be seen to range from partial surface, to near full surface coverage, indicating that the lipids are free to diffuse and explore the whole particle within the measurement time. It also shows that a single lipid can explore the whole nanoparticle during the time lapse of the measurement. Due to the inherent nature of the sample this is expected as the mobility of lipids within such layers should be relatively high and restricted only to stochastically random walks within the lipid layer. After this qualitative observation we moved to look at the quantitative mobility data. In keeping with the quality thresholds previously discussed, multiple MSD curves are found for each particle, which can vary significantly. Due to the random nature of the motion, coupled with the relevant timescale, events ranging from sub diffusion to super diffusion can be seen. However, super diffusion is most likely attributed to the inherent random nature of molecule mobility, rather than any active motion within the sample, although it may be attributed to potential thermal effects.

There is a significant heterogeneity between the particles, as shown in DOPC (A–D). This can be seen as both a variance in the amount of the response as well as the surface coverage.

Fig. 3A shows the distribution of molecule counts per nanoparticle. This shows a statistical distribution related to the random incorporation of the labeled lipid. The 3D localization clouds can also be used to fit the particle size as shown in Fig. 3B. In most cases within the DOPC system, the core was fitted quite well once the localizations last long enough to span a significant proportion of the particles surface. On the contrary for DPPC we observed highly localized tracks with molecules wiggling around a fixed position. This agrees with the gel-like nature of DPPC. Consequently, the whole NP is not explored, and the particle size cannot be extracted. Fig. 3B shows some expected polydispersity of NP size, expected from the synthesis. Interestingly Fig. 3C and D shows the results of Diffusion coefficient measurements both collectively analyzed and on particle-by-particle basis. While most of the particles have comparable D, due to the similar lipid composition, there are some variations due to stochastic variation of NP composition and some outliers, probably NPs where there are imperfections in the synthesis that strongly alter molecule diffusions.

Fig. 4 shows a quantitative comparison between the DOPC and DPPC systems. While there is a visual difference between the particles it is not obvious how this translates in motion quantitation and which parameter is more meaningful to compare. In the case of the selected particle, the area in which a lipid can traverse within the DOPC SSLB is quite substantial compared to the area the DPPC can move in. This is the most common result across all the acquired data. In general, within the DOPC system the individual lipid can be seen to move freely around the surface, whilst regional confinement is seen within the DPPC system. Surprisingly, the global MSDs measured at 0.16 μm^2^ s^−1^ ± 0.01 μm^2^ s^−1^ (DOPC) and 0.11 μm^2^ s^−1^ ± 0.01 μm^2^ s^−1^ (DPPC), for both systems are not as different as expected. Indicating that confinement is more significant than the mobility within the lipid layer. This outcome is in keeping with the selection shown in Fig. 3, where the step size (average distance between consecutive localization) is a preliminary way to show MSD and shows the lipid mobility is similar in both. In accordance with the tracking method employed by MINFLUX, the resulting positions which are recorded relate to a cartesian XYZ point for the average fluorophore location. This leads to an associated error as the fluorophore is moving on a curved surface, which can impact the calculated MSD and as such an approximation was performed to assess its impact (Fig. S1†). Found to be less than a 1% error, its impact for now can be considered low. Therefore, MINFLUX can be readily applied to assess molecules around other SSLB architectures or systems of a comparable size.

**Fig. 4:**
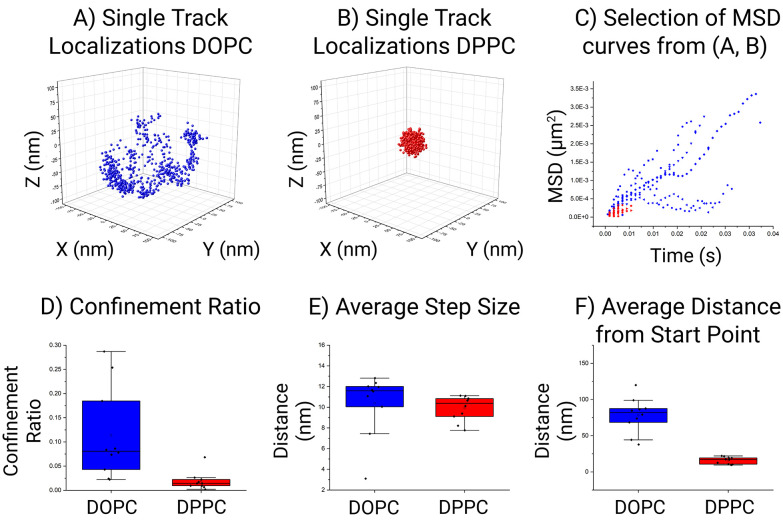
Single tracked event around a SSLB (A–C) and 10 SSLB's with the most localization analysis (D–F) within the DOPC (blue) and the DPPC (red) system. To display the relative confinement within the inherent system, the localizations from a single event are plotted around the center point of the respective localization clouds (A and B). For the first 10 instances of highest quality data yields the MSD (μm^2^) *versus* time (s) is plotted (C). The collective data from the first 10 SSLB's are combined to give the confinement ratio (D), the average step size (E) and the average distance from the start point (F).

We present the application of MINFLUX to the novel study of single molecule lipid mobility around the surface of Silica Supported Lipid Bilayers (SSLB's). MINFLUX can assess these systems and with the help of a custom developed analysis pipeline and GUI, yield results pertaining to the estimated size of the underlying Silica core, the diffusion coefficient of the lipids mobility, in addition to the further assessment of the confinement ratio of the system. We demonstrated this with a custom developed image analysis pipeline and GUI and further data analysis to extract and combine the data to yield combined results.

From the DOPC SSLB's, the fitted core was found to be 165 nm nominally. Whilst for the DPPC particles the fitting was not successful, which is due to the increased confinement found within that system. The global diffusion coefficient for a lipid coming from the filtered data from all particles within the DOPC system was found to be *ca.* 0.16 μm^2^ s^−1^ whilst it was 0.11 μm^2^ s^−1^ for the DPPC system. These values are below the known literature values for diffusion on larger and non-supported particle systems. However, literature MSD is seen to vary between 1–10 μm^2^ s^−1^, where the size of the core particle is bigger than what studied here. Furthermore, the trend is that MSD is linear with particle size, so smaller particles should yield a slower observed MSD is, as such our results are in keeping with the current trend observed. In addition, the mode of operation of MINFLUX may be biased towards slower events as fast movements can be lost.

In summary, thanks to MINFLUX we were able to track individual lipids and gain new insights into their mobility within the DOPC and DPPC silica-supported bilayers. This is a proof-of-concept showing that MINFLUX can be applied beyond biological systems and can potentially impact nanoscience and material chemistry. We envision MINFLUX to be applied in the chemistry field to answer questions still open due to the lack of techniques able to investigate materials at the nm level in wet native conditions. MINFLUX imaging's spatial resolution can be used to find molecular distributions at the nanoscale, such as ligand distribution on nanoparticles while MINFLUX tracking's temporal resolution could be a unique tool to measure dynamic molecular events such as nanoparticles surface rearrangements and binding interactions. Moreover, beyond applications in the nanoparticles field, our analysis pipeline can be used to MINFLUX imaging of other very relevant membrane-coated nanoscale samples of biological relevance such as viruses, bacteria and extracellular vesicles.

## Author contributions

L. A., L. W., T. Z., L. W. F. conceived the experiments and the project. L. W. prepared the SSLB samples, S. S. imaged the samples and discussed results, Z. H. wrote the analysis pipeline, L. W. F. analyzed data, discussed results, and wrote the paper. All authors reviewed the manuscript.

## Conflicts of interest

There are no conflicts to declare.

## Supplementary Material

NR-017-D5NR00948K-s001

## Data Availability

All code, models, and results are available on GitHub: https://github.com/EMBL-ICLM/MINFLUX-particle-tracking-and-diffusion-analysis.
