# Supplementary material for: MINFLUX: μs and nm precision 3D tracking of dynamic lipid mobility on nanoparticles
Source: Nanoscale. 2025 Jul 2;17(28):16609–15. doi: 10.1039/d5nr00948k (PMC12224226; doi:10.1039/d5nr00948k)
Supplement: NR-017-D5NR00948K-s001 [file NR-017-D5NR00948K-s001.pdf]

Supplementary Information (SI) for Nanoscale.
This journal is © The Royal Society of Chemistry 2025

Supporting Information

MINFLUX: µs and nm precision 3D tracking of dynamic lipid mobility on nanoparticles.

Laurence W. Fitzpatrick[1], Laura Woythe[1], Ziqiang Huang[2], Sebastian Schnorrenberg[2], Timo Zimmermann[2], Lorenzo Albertazzi[1].

1. Department of Biomedical Engineering, Institute of Complex Molecular Systems, Eindhoven University of Technology, Eindhoven, The Netherlands.
2. EMBL Imaging Centre, EMBL-Heidelberg, Heidelberg, Germany

Experimental Methods

Materials

Fluorescent silica NPs (Sicastar-greenF, plain) of 200 nm diameter were obtained from Micromod Partikeltechnologie GmbH. 1,2-dioleoyl-*sn*-glycero-3-phosphocholine (DOPC) and 1,2-dipalmitoyl-*sn*-glycero-3-phosphocholine (DPPC), extrusion filter supports (10mm) and PC membranes for extrusion were purchased from Avanti Polar lipids. DOPE-CAGE635 was obtained from Aberrior GmbH. 4-(2-hydroxyethyl)-1-piperazineethanesulfonic acid (HEPES) buffer (1 M) was obtained from Thermo Fischer Scientific. Sodium chloride (NaCl) was obtained from Sanal. Chloroform was purchased from Merck Life Science. Gold nanoparticles (100 nm) were purchased from Nanopartz.

Synthesis of SSLB

The synthesis of SSLB was performed as described by Giakoumatos et al[1,2]. Briefly, small unilamellar DOPC or DPPC vesicles were formed via thin film hydration and extrusion. Therefore, 195 µl DOPC or DPPC lipid stock dissolved at 10 mM in chloroform were mixed with 10 µl DOPE-CAGE635 lipid dissolved at 10 µM in chloroform (final concentration 500 nM DOPE-CAGE635) and added to a glass vial, where the chloroform solvent was evaporated under continuous vortexing and a nitrogen stream. A lipid thin film was formed on the glass vial walls after all the chloroform evaporated. The lipid film was rehydrated using 1 ml of 10 mM HEPES and 50 mM NaCl buffer (pH 7.4) that was heated above the melting temperature of the lipids (25°C for DOPC and 60°C for DPPC) and vortexed for 2 minutes. The resulting vesicle suspension was extruded using an Avanti Mini Extruder 21 times through a 200 nm filter, 21 times through a 100 nm filter and 21 times through a 50 nm filter above the respective melting temperatures of the lipids. For DPPC vesicles, the final extrusion step with the 50 nm filter was performed for 41 extrusion steps. Next, SSLB were formed the same day by mixing 400 µl of vesicle suspension, 20 µl of Sicastar Silica Nanoparticles (stock 50 mg/ml) and 980 µl 10 mM HEPES and 50 mM NaCl buffer for 1 h above the melting temperature of the lipids under shaking in a ThermoMixer®. Finally, SSLBs were washed thrice via centrifugation at 16000 g for 15 minutes to remove free lipid vesicles and redispersed in 10 mM HEPES and 50 mM NaCl. SSLB were stored at 4°C and used within one week.

MINFLUX imaging

SSLBs were imaged on an ibidi 8-well glass bottom µ-Slide. For active sample stabilization, 200 nm spherical gold nanoparticles (Nanopartz, #A11-200-CIT-DIH-1-10) were added to the imaging well. MINFLUX tracking was performed on an Abberior MINFLUX microscope (Abberior, Goettingen, Germany) equipped with a Olympus 1.4 NA 100× Oil objective lens as previously described[3]. Single particle tracks were acquired in MINFLUX 3D-tracking mode using a 642 nm excitation laser (21.8 µW). Laser powers were measured at the position of the objective back focal plane using a Thorlabs PM100D power meter equipped with a S120C sensor head. The imaged fields of view were approximately 20x20 µm, with an average duration of 30-45 minutes for one measurement. Valid localizations were recorded when a minimum of 100 photons in the last iteration were detected. Localizations were considered invalid and filtered out when a second fluorophore was detected in the same detection volume and when the fluorophore failed to be localized in 4 consecutive probing steps. Analysis of the collected localizations was performed using a custom MATLAB script.

MINFLUX Analysis

We used a custom data processing and analysis workflow to analyze the lipid mobility on silicon nanoparticles. Trajectories from fluorescent molecules are pre-processed and reconstructed from MINFLUX data, to quantify the underline diffusion properties, as a readout of the lipid mobility. The pre-processing, reconstruction, and visualization of the MINFLUX tracking data are all done through custom written MATLAB scripts and GUI tools.

In the first pre-process step, we performed a set of filtering to the MINFLUX raw data to remove noise and data that was not suitable for processing. We start with the raw data that was exported to MATLAB data (.mat) format. MINFLUX gathered tracking results into groups and assigned unique ID to each track, denoted as 'tid'. The 'tid' attribute is assigned to every localization as part of the raw data. Therefore, we first extracted 3D coordinates, their time stamp, and associated 'tid' of all valid localizations from MINFLUX raw data for pre-processing.

The pre-processing on the raw data basically consists of two steps: A first filtering step, followed by a clustering step.

We did little spatial filtering on the localization data, by removing data located near the border (within 1% of the border) of the XY field of view. To ensure the completeness of tracking data at this stage, the filtering is on track level. It means if any data point falls into the 1% marginal region, an entire track containing that data point is discarded.

At this stage, we also correct for refractive index mismatch[4,5] (RIM), to compensate for the axial aberration. To do this, we measured and equalized the spatial spread in each axis for every track. For a given trace, we compute first the interquartile range (iqr) between 25% and 75% percentile of X, Y, and Z axis of the data. And then we calculate the ratio of XY geometrical mean over Z iqr value, as the RIM score for each track. Finally, we calculated a weighted mean of the RIM scores from all the tracks, based on the number of data points within each track, to generate the final RIM correction factor. The RIM correction factor is measured on a daily-bases and applied to all tracking experiment in the same day. For the 3 consecutive days of imaging, we measured the RIM correction factors as 0.6232, 0.6388, and 0.6237 (Fig.3), indicating rather low variation of the axial aberration across days of imaging.

Each MINFLUX tracking process takes roughly several tens of data point to stabilize. As a result, a lot of tracks exhibit a short tail-like portion in the beginning which reflects this initial targeting process. A given track ends normally when the tracking process slowly loses the being tracked molecule, which resulting in also relatively low-quality data at the end of tracks. Therefore, for each track we discarded 100 data points both from the beginning and at the end of the track. By visual inspection, this filtering procedure effectively removed the 'tail-like' portion of each track. Shorter tracks that contain no more than 200 data points are discarded at this step.

The time stamps for each localization were exported with effective precision in the range of several tens of microsecond. We extracted time intervals as the increment between time stamps of two adjacent localization events and rounded it to 0.1 millisecond (ms) precision for the MSD computation and diffusion analysis of this study.

After filtering step, we then spatially clustered the processed data with density-based scan (DBSCAN). We make use of the track identity of the data, to cluster on the track level, rather

than individual localization data points. This way, the clustering process has been speeded up significantly, while the computation workload has greatly reduced. We first calculated the centroid coordinates of all tracks. Then all the centroid coordinates are clustered such that any pair of track centroids located within a radius of 200 nm to each other will be clustered together. A cluster ID is then assigned to each data point, similarly as the track ID. Naturally, data belonging to the same track also belongs to the same cluster.

We then fit a spherical shell to the clusters with the least square method. This fitting approach minimizes the residual sum of squared distance (error) of all points to the sphere surface. If the fitting error is too large, or the fitting result deviates much from expected geometry (e.g.: points form a plane rather than a 3D spherical surface), the fitting is considered failed. Clusters with failed fitting are marked but kept for down-stream diffusion analysis. This is because when two or more silicon cores are close to each other, nanoparticles imaged on their surface sometimes cannot be distinguished by spatial clustering. Such cases would result in a cluster with failed sphere fitting, but still contains meaningful data that can be visually categorized and further analyzed. Nevertheless, successful fitting results are also marked, and better fitting results with smaller fitting errors are sorted more to the front as good candidate for further inspection and analysis.

To analyze diffusion behavior, we compute mean squared displacement (MSD) and diffusion coefficient for each cluster. As mentioned above we extracted time interval (dt) between each adjacent localization and inspected its property. It is obvious that the time intervals from MINFLUX tracking experiment were not always consistent, and instead roughly segregated into several different levels (fig. 2E). These levels are in fact correspond to the 1, 2, 3, or more rounds of MINFLUX beam pattern scanning on the tracked molecule. Since more rounds of scanning are made only when previous round(s) failed to locate the molecule, larger time interval is also normally associated with larger uncertainty in localization. To account for this, we break a complete track from previous filtering steps into shorter track segments, around these large time intervals. We define a breaking criterion that only allows maximum 2 rounds of scanning within a given track segment. This is implemented by estimating the base level time interval minDt, which corresponds to only 1 round of scanning. We then set a threshold to the time intervals as 2.5 * minDt, to be the breaking criterion. This proven to be sufficient to effectively distinguish between 2 and 3 rounds scanning in the data. The track segments from all tracks belonging to a given cluster are stored and used in subsequent MSD computation.

To compute the MSD associated with each unique time interval, we used a modified version of msdanalyzer (https://tinevez.github.io/msdanalyzer), a designated MATLAB class to perform MSD computation and analysis. The main advantage of this package is it can deal with non-equidistant time intervals, which is better suited for our MINFLUX tracking data. We adopted a vectorized approach to save CPU time for the MSD computation in MATLAB, at the expense of RAM. With our code, the memory required to process a complete track segment consisting of ~ 40k data points would be roughly 128 Gb, as tested on a Windows 10 machine with MATLAB 2023b. However, given the average time interval from MINFLUX tracking data is in the range of several hundreds of microseconds, we haven't encountered so far, any complete track segments contain more than 10k data points. In addition, for the objective of this study, we are mainly interested in the fast component of the diffusion behavior and as a result would not necessarily need the MSD computed from such long track segments. For each

track segment extracted in the last step, all possible time intervals, dt, are calculated. Then MSD values corresponding to each unique dt values are computed. The MSD and dt pairs are computed and stored for each track segment. We also computed cluster-wise weighted average values, with the same procedure that is described in msdanalyzer. Shortly paraphrasing, *the weights are taken to be the number of averaged delay* (time interval)*, which favors short delays*.

The diffusion coefficient can be calculated for each pair of MSD and dt as *D = MSD / (2 * ndim * dt)*, with *ndim* being the dimensionality of the trajectory. Therefore, in the 3D tracking case, diffusion coefficient D is thus calculated as *MSD / (6 * dt)*.

The analysis results from the above filtering, clustering, sphere fitting, and MSD analysis steps are all stored and exported to MATLAB data file format. The result file can be loaded into MATLAB for data visualization and further analysis, or to be loaded with the custom data visualization tool that is created together with the analysis scripts.

To facilitate data visualization and further analysis, a GUI tool (Fig S.4) which generates MATLAB figures is also created, as part of the analysis workflow (Fig. 2). The first overview figure displays coordinates of the processed data as a 3D scatter plot. It enables data load, coloration, and data selection through custom designed buttons (Fig. 2A). The 'color' toggles between 3 different color modes of the scatter plot: colored by track, colored by cluster, or colored by selected cluster, that is displaying in a second cluster view figure. The cluster view figure shows the selected cluster as a 3D scatter plot and uses different colors to differentiate different tracks belonging to the same cluster. If the sphere fitting was successful, a semi-transparent sphere shell is generated based on the fitting parameters and overlay with the scatter plot (Fig. 2B). We made 6 subplots to further display the properties of the tracking data and tracking result. The 6 subplots are gathered in 2 groups: the top 3 subplots show displacement, velocity, and time-intervals against time; the bottom 3 subplots show the MSD vs. time interval plot, histogram of the diffusion coefficient, and cumulative frequency of the diffusion coefficient. A time slider is also implemented to highlight tracking data and values (in the top 3 subplots) corresponding to the slider indicated time point.

References

(1) Woythe, L.; Porciani, D.; Harzing, T.; van Veen, S.; Burke, D. H.; Albertazzi, L. Valency and Affinity Control of Aptamer-Conjugated Nanoparticles for Selective Cancer Cell Targeting. *Journal of Controlled Release* **2023**, *355*, 228–237. https://doi.org/10.1016/j.jconrel.2023.01.008.

(2) Giakoumatos, E. C.; Gascoigne, L.; Gumí-Audenis, B.; García, Á. G.; Tuinier, R.; Voets, I. K. Impact of Poly(Ethylene Glycol) Functionalized Lipids on Ordering and Fluidity of Colloid Supported Lipid Bilayers. *Soft Matter* **2022**, *18* (39), 7569–7578. https://doi.org/10.1039/D2SM00806H.

(3) Schmidt, R.; Weihs, T.; Wurm, C. A.; Jansen, I.; Rehman, J.; Sahl, S. J.; Hell, S. W. MINFLUX Nanometer-Scale 3D Imaging and Microsecond-Range Tracking on a Common Fluorescence Microscope. *Nat Commun* **2021**, *12* (1), 1478. https://doi.org/10.1038/s41467-021-21652-z.

(4) Huang, B.; Wang, W.; Bates, M.; Zhuang, X. Three-Dimensional Super-Resolution Imaging by Stochastic Optical Reconstruction Microscopy. *Science* **2008**, *319* (5864), 810–813. https://doi.org/10.1126/science.1153529.

(5) Thevathasan, J. V.; Kahnwald, M.; Cieśliński, K.; Hoess, P.; Peneti, S. K.; Reitberger, M.; Heid, D.; Kasuba, K. C.; Hoerner, S. J.; Li, Y.; Wu, Y.-L.; Mund, M.; Matti, U.; Pereira, P. M.; Henriques, R.; Nijmeijer, B.; Kueblbeck, M.; Sabinina, V. J.; Ellenberg, J.; Ries, J. Nuclear Pores as Versatile Reference Standards for Quantitative Superresolution Microscopy. *Nat Methods* **2019**, *16* (10), 1045–1053. https://doi.org/10.1038/s41592-019-0574-9.

Supplementary Figures

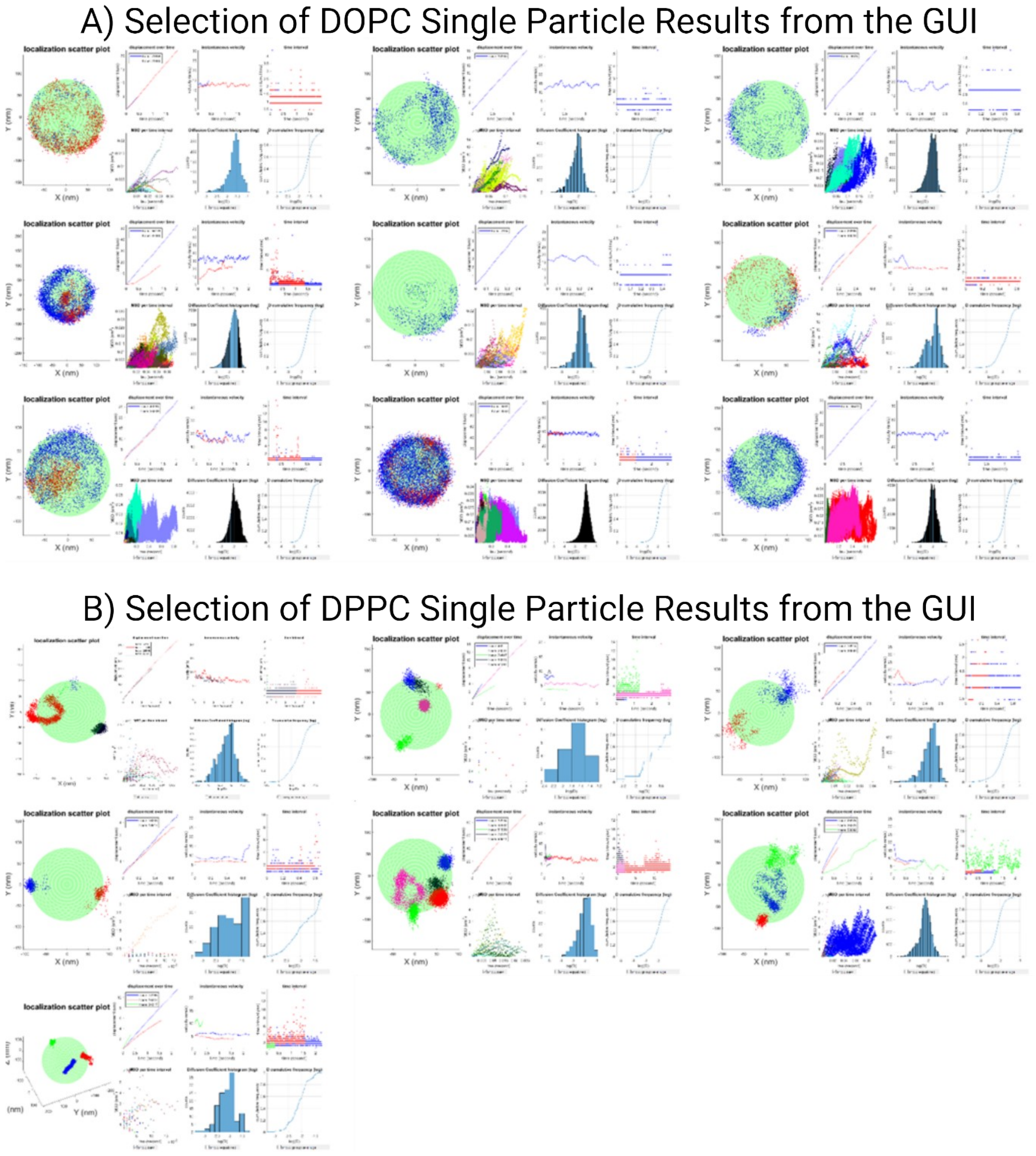

Fig S.1: Multiple Si-DOPC SSLB systems (A) and Si-DPPC SSLB (B) systems as displayed within the custom GUI, showing both intra and inter particle variance. Furthermore, showing the difficulty to correctly identify the underlying Silica particle, due to the lack of mobility within the DPPC to traverse the full particle surface.

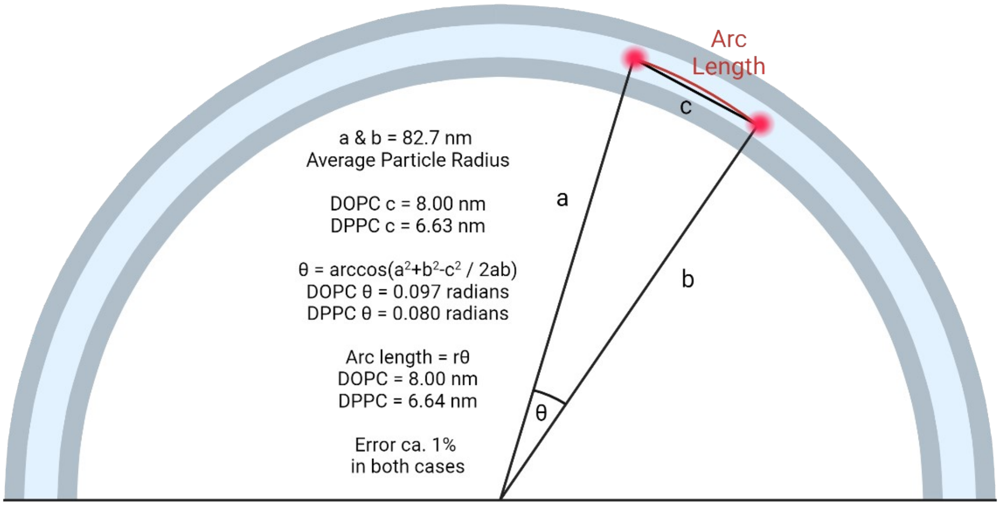

Fig S.2: The distance a tracked fluorophore moves between consecutive frames is measured as a point-to-point distance in 3D cartesian space, however systematically it is moving in 3D on a curved surface. As such there is an associated error between the measured distance c and the arc length which it moved on. To find this arc length, the average global step from the MSD is mapped onto the average radius of an acquired particle, from which the corresponding arc length is obtained. Comparing the arc length to the cartesian distance gives the associated error within the calculated MSD, which is found to be ca. 1%.

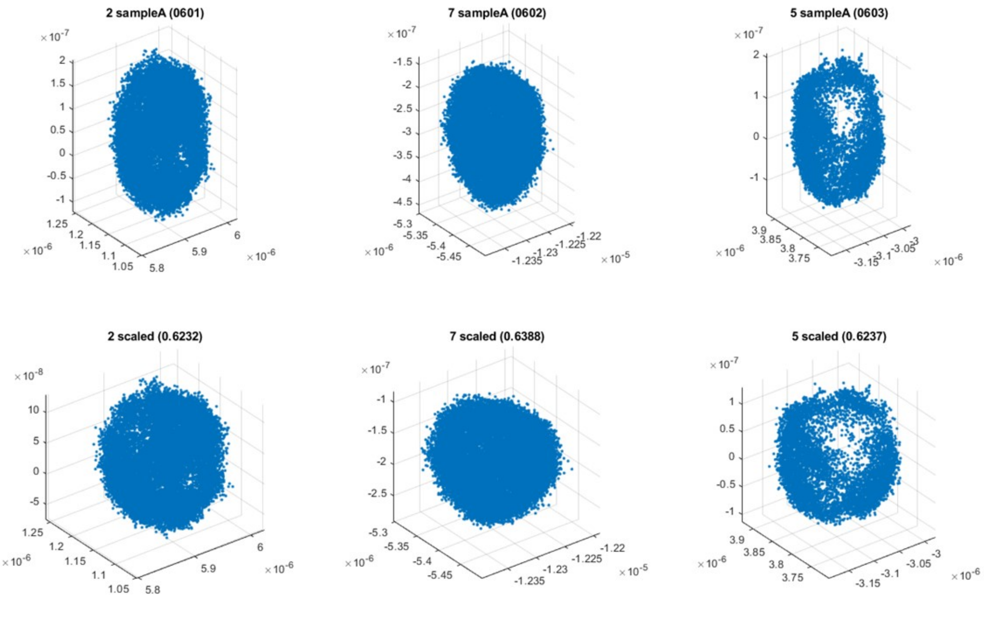

Fig S.3: Refractive index mismatch correction: upper row, scatter plot of selected track for each day of acquisition; lower row, the same data after refractive index mismatch correction (z coordinate scaled by RIM correction factor).

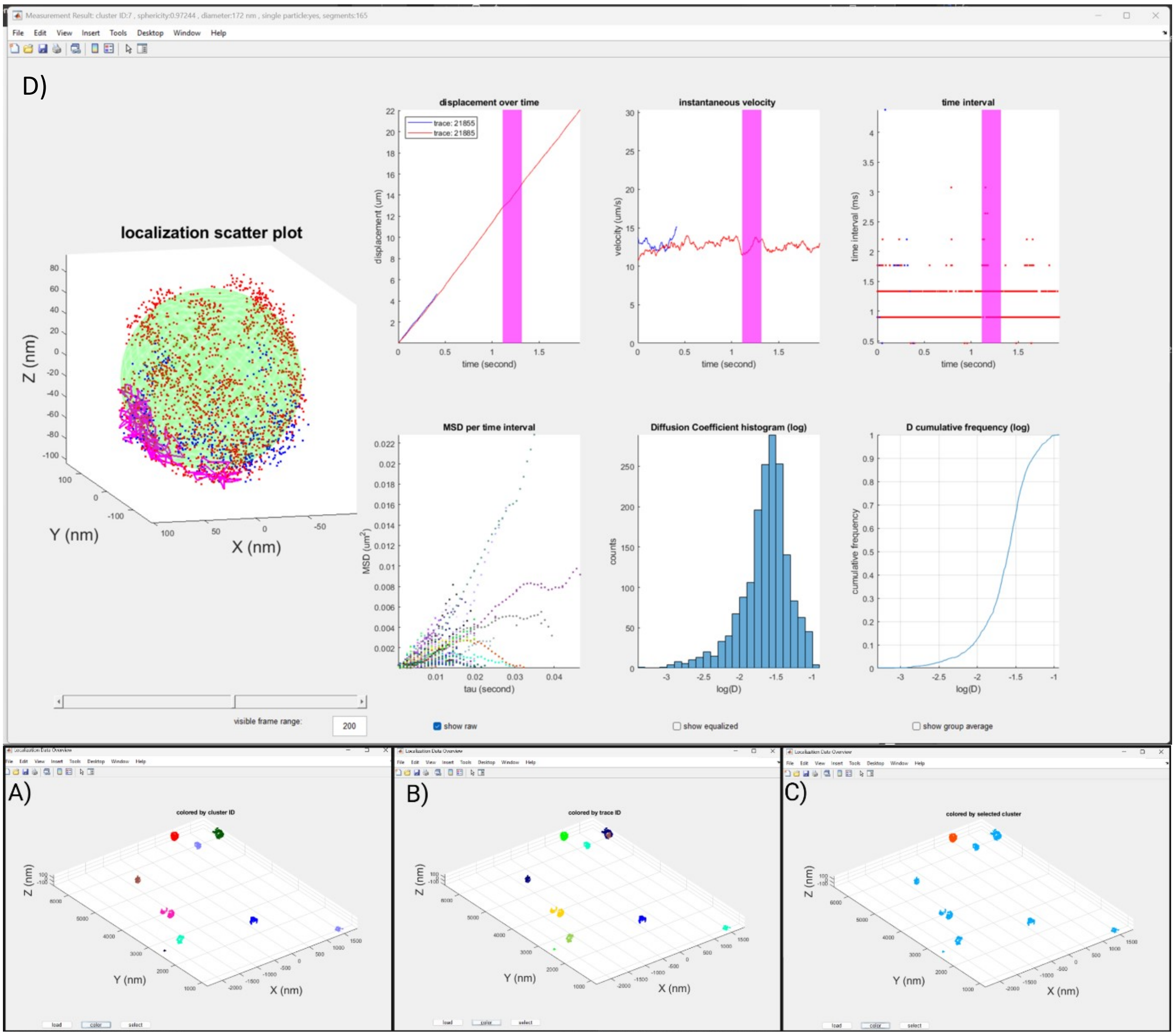

Fig S.4: GUI demonstration. First the dataset is presented in the full area encompassing multiple particles. At this point the data is already clustered by DBSCAN, and the user can choose between three different visualizations: A) by cluster ID, i.e. the software will color-code with the same color all tracks belonging to the same nanoparticle; B) by trace ID, i.e. the software will assign to each track a different color. These two visualizations will help the user to choose a nanoparticle of interest. Our suggestion is to start with the trace ID visualization to assess the number of tracks per NP (ideally you want as many as possible to have more sampling) and then switch to the cluster ID to select the cluster of interest. Once a cluster is selected the interface show the cluster selected in red and the others in blue (see panel C). Once selected the cluster its analysis is highlighted (D). In this window the use can appreciate in the left panel the spatial distribution of the localization (color

coded per track) and the fit sphere in green. On the right the user can find six plots of relevant properties: i) displacement versus time, ii) instantaneous velocity; iii) time intervals; iv) Mean square displacement and v-vi) diffusion coefficient both in histogram and cumulative distribution. These provide a broad overview of the diffusion properties of the NP selected. As an extra feature it is possible to highlight only a part of the trajectory (pink selection in panel D) and correlate the spatial position of such localization on the left with the measured parameters on the right. This feature is very useful when a trajectory is not homogenous over time (e.g. change velocity due to a binding event or encounter an area of the sample with different properties)
